# Magnetomyographic evaluation of motor unit size that is robust to changes in distance to sensor

**DOI:** 10.1093/braincomms/fcaf294

**Published:** 2025-08-20

**Authors:** Tai Otani, Miho Akaza, Shigenori Kawabata, Hirokazu Natsui, Taishi Watanabe, Yuki Miyano, Ryoichi Hanazawa, Yoshiaki Adachi, Kensuke Sekihara, Tadashi Kanouchi, Takanori Yokota

**Affiliations:** Department of Neurology and Neurological Science, Graduate School of Medical and Dental Sciences, Tokyo Medical and Dental University, Tokyo 113-8519, Japan; Department of Clinical Information Applied Science, Graduate School of Medical and Dental Sciences, Tokyo Medical and Dental University, Tokyo 113-8519, Japan; Department of Advanced Technology in Medicine, Graduate School of Medical and Dental Sciences, Tokyo Medical and Dental University, Tokyo 113-8519, Japan; Department of Neurology and Neurological Science, Graduate School of Medical and Dental Sciences, Tokyo Medical and Dental University, Tokyo 113-8519, Japan; RICOH Futures BU, RICOH Company, Ltd., Tokyo 143-8555, Japan; RICOH Futures BU, RICOH Company, Ltd., Tokyo 143-8555, Japan; Department of Clinical Biostatistics, Graduate School of Medical and Dental Sciences, Tokyo Medical and Dental University, Tokyo 113-8519, Japan; Applied Electronics Laboratory, Kanazawa Institute of Technology, Nonoichi 921-8501, Japan; Department of Advanced Technology in Medicine, Graduate School of Medical and Dental Sciences, Tokyo Medical and Dental University, Tokyo 113-8519, Japan; Department of Laboratory Medicine, Graduate School of Medical and Dental Sciences, Tokyo Medical and Dental University, Tokyo 113-8519, Japan; Department of Neurology and Neurological Science, Graduate School of Medical and Dental Sciences, Tokyo Medical and Dental University, Tokyo 113-8519, Japan

**Keywords:** magnetomyography, superconducting quantum interference device, spatial filtering method, motor unit size, neurogenic change

## Abstract

In clinical practice, motor unit (MU) size is evaluated using needle electromyography to diagnose the cause of muscle weakness, whether myogenic or neurogenic. However, needle electromyography is influenced by the conductance of the muscle tissue and the distance from the MU to the electrode. In contrast, the magnetic field generated by a skeletal muscle is not distorted by subcutaneous tissues because their magnetic permeability is considered equal to that of free space. Therefore, we hypothesized that MU amplitude can be measured via magnetic field recordings. We tested this hypothesis by recording MU activity in the abductor pollicis brevis muscle. We then evaluated the MU size difference between healthy individuals and patients with spinal muscular atrophy and spinal-bulbar muscular atrophy. Furthermore, we assessed whether our method could consistently evaluate the MU size regardless of the sensor-muscle distance. Myomagnetic fields of single MUs evoked by electrical stimulation of the median nerve were measured. We used a biomagnetometer equipped with 132-channel superconducting quantum interference device sensors orientated upwards and arrayed on a quasi-planar surface. We applied a spatial filtering method that can estimate the current distribution from the magnetic field even for a conductor with unknown configuration or conductivity distribution and that does not require an a priori assumption of how many source currents are present. We visualized the electrical activity of 12 MUs of the abductor pollicis brevis muscle from eight healthy individuals and of two MUs from two patients with spinal muscular atrophy and spinal-bulbar muscular atrophy. Four current patterns were identified in the MU electrical activity. In all MUs, current towards the innervation zone was observed just after the start of activities. We called this current ‘initial muscle-directing current’. At the same time, currents directed proximally and distally from the middle of the muscle were observed in most MUs. Initial muscle-directing current was more than 3 or 10 times larger in patients with spinal muscular atrophy and spinal-bulbar muscular atrophy than in healthy individuals. Initial muscle-directing current was estimated to weaken by 5.5% for every 5-mm increase in distance from the sensor array. Initial muscle-directing current is considered to reflect the activity near the neuromuscular junction and can be an index of MU size. The results confirmed our hypothesis that MU amplitude can be evaluated using magnetic measurements. This novel and non-invasive magnetomyography method can evaluate MU size with little influence of distance and has the potential to supersede needle electromyography.

## Teaser

Otani *et al*. estimated motor unit current from the magnetic field and showed four current patterns that were much larger in patients with neurogenic disease than in healthy individuals. This method was robust to changes in sensor distance and allowed visualization of the initial current triggering subsequent motor unit activity.

## Graphical Abstract

**Graphical Abstract:**
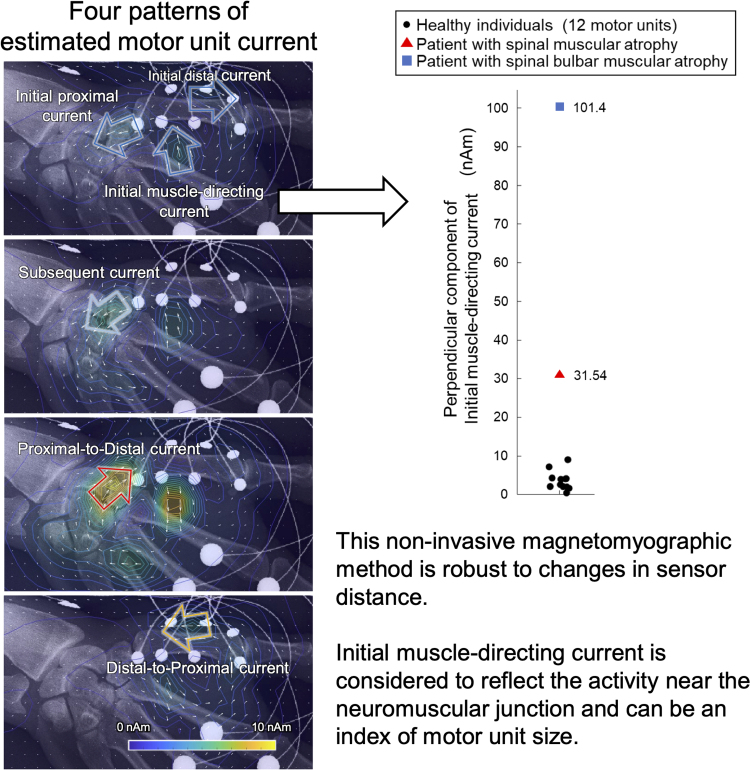

## Introduction

A motor unit (MU) is a functional unit that comprises a motor neurone and the muscle fibres that it innervates. A motor neurone signal triggers the simultaneous contraction of all of the muscle fibres within that MU.^1^ Motor neurone damage causes denervation of muscle fibres, which are then reinnervated by the remaining motor neurones. This increases the number of muscle fibres per MU. In contrast, in myogenic disorders, the number of muscle fibres belonging to a single MU is reduced. Therefore, when the causes of muscle weakness or atrophy are being considered, an increase or decrease in the number of muscle fibres or in the MU size can be a determinant of whether the cause is neurogenic or myogenic.^2^

In clinical practice, needle electromyography (nEMG) is used to obtain intramuscular recordings and evaluate MU potentials (MUPs). MUP variables such as potential amplitude, duration, area, shape and interference pattern are assessed to determine whether a neurogenic or myogenic change is present.^3^ The needle electrode can be inserted close to firing muscle fibres and has a limited pick-up area corresponding to a 2.5-mm radius from the needle tip,^4^ allowing the differentiation of each MUP. However, the limited pick-up area of the needle electrode leads to variations in the amplitude and waveform of the recorded MUP based on the positional relationship between the MU and the needle tip.^5^ Thus, the examiner must be skilled to objectively evaluate the MU size in nEMG. It is also an invasive and painful procedure, which limits its clinical use, particularly in children and in follow-up studies.

Surface electromyography, which records electrical potentials from the surface of the skin, is non-invasive. However, it is difficult to use this technique to evaluate a single MU with several electrodes. This is because surface electromyography has a large pick-up area that records multiple MUPs. In addition, the waveforms recorded from the surface lose their high-frequency components, resulting in waveforms from different MUs becoming similar to each other.^6^ Some studies have attempted to differentiate between myogenic and neurogenic changes without evaluating a single MUP. The MU number estimation method provides a framework for estimating the number of motor units (MUs),^7-9^ within which the MU number index method offers a specific approach to obtain an index reflecting the MU number.^10,11^ Finally, the clustering index method evaluates the coherence of MUPs in voluntary contractions.^12,13^ These methods use several electrodes, while other studies have applied mathematical processing to decompose single MUPs from waveforms containing many MUPs recorded using multichannel high-density surface electrodes,^14,15^ estimate the number of MUs,^16^ and evaluate the firing rate of MUs.^17,18^ Drost *et al.*^19^ discriminated the MU size between healthy individuals and post-polio patients by evaluating the decomposed MUP waveforms, but this method is not widely used. Ultimately, potential recording is theoretically affected by the conductivity of the subcutaneous tissue and the distance between electrode and electrically active muscle, which can alter the MUP waveform.

In contrast, the magnetic field associated with current in the muscle is not distorted by the condition or shape of the surrounding tissue because the magnetic permeability of any living tissue is considered equal to that of free space.^20^ Wikswo *et al.*^21^ indicated that the absolute strength of the muscle current can be evaluated. Human magnetomyography was first performed by Cohen^22^ around the elbow and near the palm during voluntary contraction with a single-channel superconducting quantum interference device (SQUID) in a shielded room. Masuda *et al.*^23^ measured biomagnetic fields of single MUs from the vastus lateralis and vastus medialis muscle, using a 64-channel SQUID magnetometer that was designed to conduct magnetoencephalography. The intensity of current moments associated with MU activities was estimated based on the equivalent current dipole method. However, the shape of the magnetometer for magnetoencephalography limits the areas of muscles that can be measured. In addition, the equivalent current dipole method based on the Sarvas formula can be adapted only to a conductor with a spherical shape or a semi-infinite plane.^24^ Therefore, it is difficult to adapt this method to hand and foot muscles, where the conductivity and the shape around the muscle are complex. Although Llinás *et al.*^25^ successfully visualized the muscle activity of the hand with magnetic field measurements and frequency-pattern analysis, the MU size was not quantified.

Because the muscle activity-associated magnetic fields observed outside the body surface are extremely small, they are conventionally detected by magnetic sensors using SQUIDs. In recent years, sensors such as optical pumping magnetometers have been developed that are expected to reduce the size and running cost. Several groups have reported basic recording studies of the magnetic field associated with muscle activity,^26-30^ but no recordings of normal MU activity have been made. Presently, SQUID sensors are the best sensors in terms of sensitivity and frequency bandwidth.^31^

Previously, our group developed a SQUID magnetometer arrayed on a quasi-planar surface to detect spinal or peripheral nervous activities.^32^ The use of the spatial filtering method enabled us to estimate the current distribution around the spinal cord and the peripheral nerves where the surrounding volumetric conductors have a complex configuration and conductivity distribution.^33^ Using our magnetometer and spatial filtering method, the intra-axonal and inward volume currents near the depolarization site can be visualized.^34-46^

In the present study, using this device and the spatial filtering method, we attempted to observe the electrical muscle activity of a single MU of the abductor pollicis brevis (APB) muscle in the hand, which has a complex configuration and conductivity distribution. Then, we compared MU size between healthy individuals and patients with spinal muscular atrophy (SMA) and spinal bulbar muscular atrophy (SBMA) (Experiment 1). Furthermore, we investigated whether our method was able to consistently evaluate MU size without being affected by the distance from the sensor array to the muscle, in contrast to nEMG (Experiment 2).

## Materials and methods

### Biomagnetometer system

**Figure 1:**
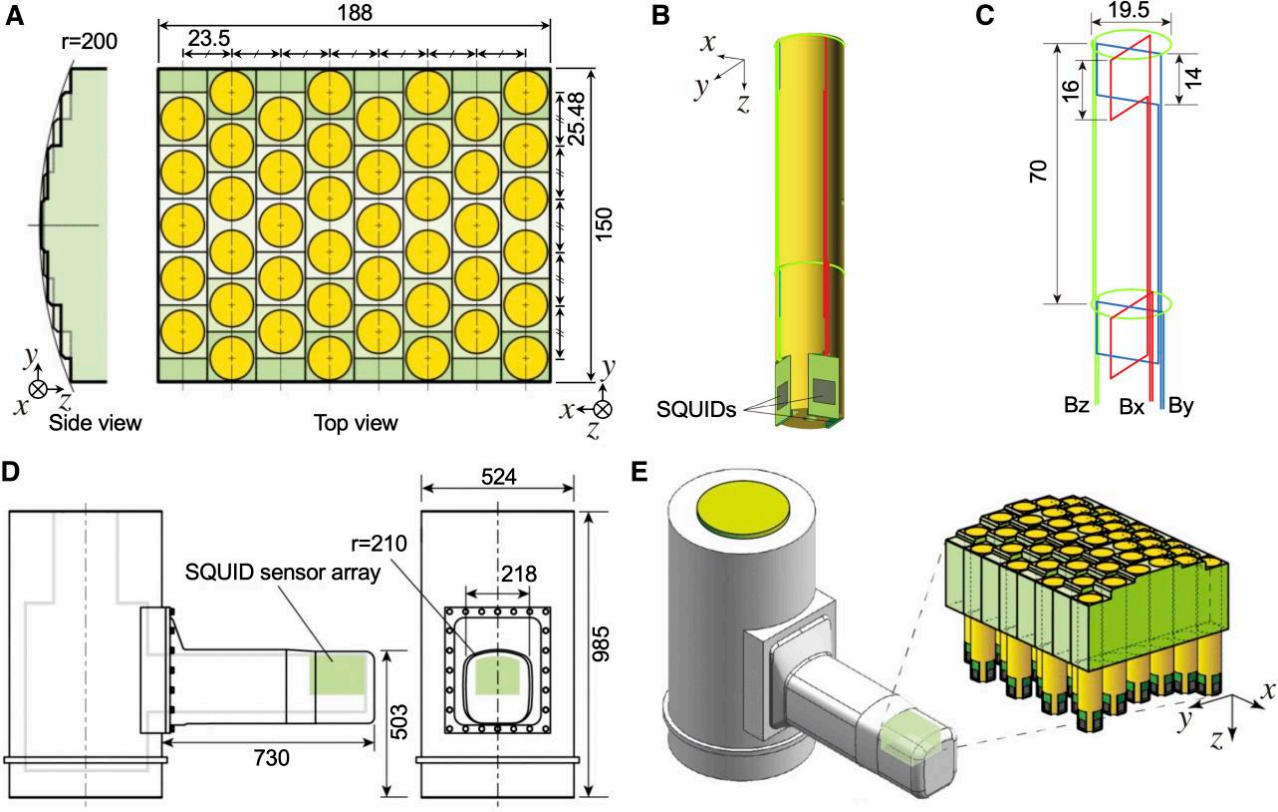
**Appearance and structure of the biomagnetometer system.** (**A**) Dimensions of the sensor array. (**B**) Schematic of a vector-type superconducting quantum interference device (SQUID) flux sensor. (**C**) Configuration and dimensions of the gradiometric pick-up coils of a vector-type SQUID flux sensor. (**D**) Dimensions of the cryostat. (**E**) Appearance of the cryostat and the sensor array. All units are in mm. r: radius of curvature. This figure was adapted with permission from Adachi *et al*.^32^ DOI: 10.1109/TASC.2021.3056492. licenced under CC BY 4.0.

All magnetic recordings were performed using a multichannel SQUID biomagnetometer system developed by the Applied Electronics Laboratory, Kanazawa Institute of Technology and RICOH Co. Ltd.^32^ The device is equipped with an array of 44 SQUID magnetometers arranged in an area of 188 mm × 150 mm along a cylindrical surface with a 200-mm radius, as shown in Fig. 1A. Vector-type SQUID magnetometers, which simultaneously detect three orthogonal components of magnetic fields (Fig. 1B), were applied to extract as much magnetic field information from the observation area as possible. The coils to pick up magnetic fields were wound around a single bobbin and oriented perpendicular to each other, as shown in Fig. 1C. A cryostat was designed to maintain the SQUID sensors in their superconducting state (Fig. 1D). The cryostat has a cylindrical main body to store liquid helium and a protrusion from its side surface. The sensor array was installed oriented upwards along the upper surface of the protrusion to detect the biomagnetic field from an object placed on the protrusion. The cool-to-warm separation in the sensor array area was 12–14 mm. Magnetic field measurements were performed in a magnetically shielded room to protect against environmental magnetic noise.

### Evoking motor unit activities and measurement of the potentials

**Figure 2:**
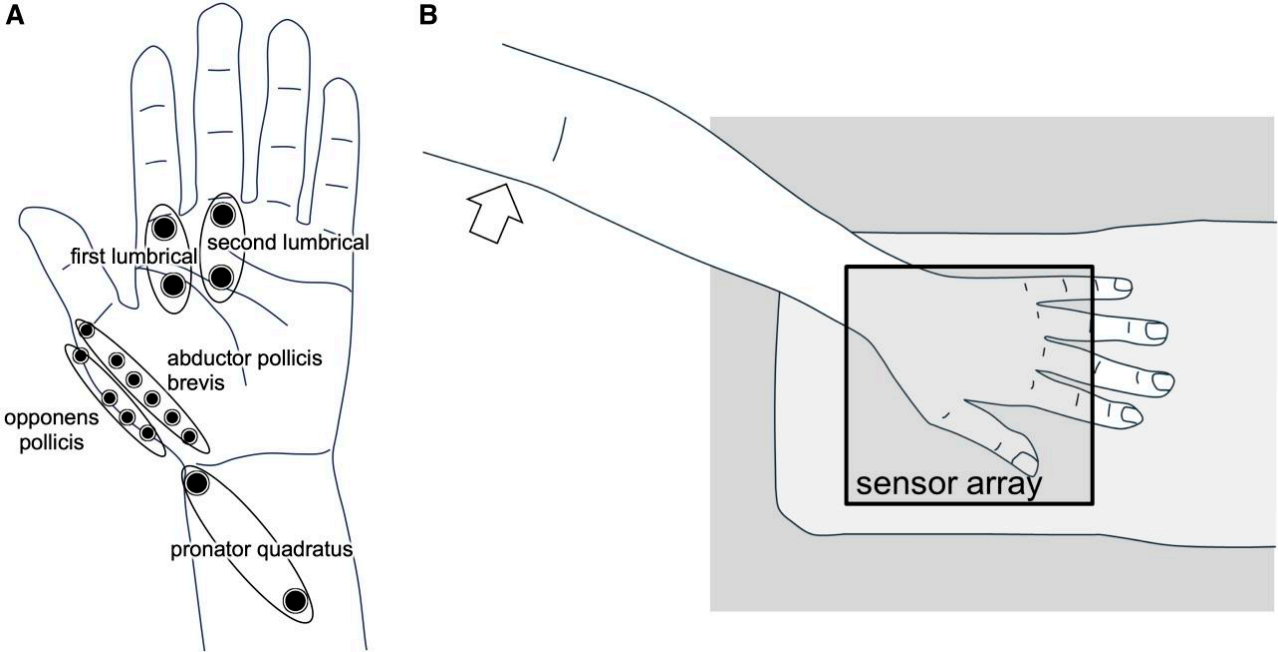
**Placement of surface electrodes.** (**A**) Surface electrodes placed on the abductor pollicis brevis, opponens pollicis, first lumbrical, second lumbrical and pronator quadratus muscles, which are innervated by the median nerve. Multiple electrodes were placed at 1-cm intervals on the belly of the abductor pollicis brevis and opponens pollicis muscles using the tendon as a reference. These electrodes had an outer diameter of 6 mm. Cup electrodes with a diameter of 10 mm were placed on the first lumbrical, second lumbrical and pronator quadratus muscles in the belly-tendon method. (**B**) Measurement position of the hand. The rectangle indicates the position of the sensor area. The median nerve was electrically stimulated between the elbow and the axilla (arrow).

A very weak electrical stimulation was applied to the median nerve between the elbow and the axilla to evoke only a single MU of the APB muscle. To reduce stimulation artefacts, the electrical stimulation site was positioned away from the magnetic sensors. The stimulus duration was 0.05 ms. The intensity was gradually increased from 0 mA and the first potential to appear was recorded as a single MUP following the ‘all-or-none law’, under the same concept as in the MU number estimation method.^7,8^ To confirm the activation of a single MU of the APB muscle, we monitored waveforms of the potential with surface electrodes placed on the APB, opponens pollicis, first lumbrical, second lumbrical and pronator quadratus muscles, which are innervated by the median nerve. Multiple electrodes were placed at 1-cm intervals on the belly of the APB and opponens pollicis muscles to identify the innervation zone. We observed the waveform based on the potential difference between adjacent electrodes placed on the APB muscle. The electrode at which the polarity of the waveform reversed was considered the innervation zone.^47^ If no polarity reversal was observed, the terminal electrode was considered the innervation zone. These electrodes had an outer diameter of 6 mm (DP-101-1500AD; Unique Medical Co. Ltd., Komae, Japan). Furthermore, cup electrodes with a diameter of 10 mm were placed on the first lumbrical, second lumbrical and pronator quadratus muscles in the belly-tendon method (Fig. 2A). An MEB2312 Neuropack device (Nihon Kohden, Tokyo, Japan) was used to record muscle action potentials and stimulate the nerve. The potentials were recorded with a 10,000-Hz sampling rate and 5–3000-Hz bandpass filter. We output the electrical stimulation trigger signals and potential waveforms from this device and input them into the biomagnetometer system for synchronization.

### Ultrasonographic measurement of the depth of the muscle belonging to the motor unit

The depth from the skin surface to the muscle belonging to the MU of interest was measured using ultrasonography because calculation of the current distribution and its intensity from the magnetic field requires the distance to the muscle. An echo probe was applied where the electrode had shown the largest amplitude in the APB muscle. While ensuring that the same MU waveforms were evoked in the remaining montages, the movement of the muscle was observed with ultrasonography. The depth of the area of muscle movement synchronized with the stimulation was measured. All ultrasonographic images were obtained using a portable B-mode ultrasound device (LOGIQ e Premium; GE Healthcare Japan Corporation, Hino, Japan) equipped with a 22-MHz linear-array probe.

### Measurement of magnetic fields

Participants were seated in a relaxed state with the palm placed over the sensor array (Fig. 2B). The forearm was lightly secured with tape. To evoke single MU activities, very weak electrical stimulation at 3 Hz was applied, as described above. The magnetic fields generated by the MU activities were measured by the biomagnetometer system using a 40-kHz sampling rate and 10–5000-Hz bandpass filter. We recorded 100–500 responses for each targeted MU.

### Signal processing and current estimation

This method may fail to evoke the MU of interest and/or evoke another MU. Therefore, we extracted only the magnetic fields corresponding to the single APB MU activity by confirming the presence of the MU activity of interest in the potential waveforms simultaneously recorded by the surface electrode. In other words, responses where other MUs were activated, where no activation was evoked, or where voluntary contraction was present were excluded. Then, the 5–500 extracted responses were added and averaged to improve the signal-to-noise ratio. A method to suppress artefacts from electrical stimulation^48^ was also applied. This algorithm first projects the columns of the measured data matrix onto the inside and outside of the spatial-domain signal subspace, creating a set of two pre-processed data matrices. The intersection of the row spans of these two matrices is estimated as the time-domain interference subspace. The original data matrix is projected onto the subspace that is orthogonal to this interference subspace. Current estimation points were placed in a grid at 5-mm intervals in a two-dimensional plane containing the MU, and the current distribution in this plane was estimated from the magnetic field using a spatial filtering method (unit-gain constraint recursively applied null-steering spatial filtering).^33,49^ The lead-field matrix was calculated based on the Biot-Savart law, using current elements placed at the estimation points as the signal sources. The distance between the MU and the sensor array was determined by the depth from the skin surface to the MU and the position of the skin surface, which was measured by marker coils.^50^

### [Experiment 1] analysis of the motor unit activity of the abductor pollicis brevis muscle

This experiment involved eight healthy individuals without abnormal neurological findings (seven men and one woman; 29–57 years of age; median age, 33.5 years) and two patients. Patient 1 was a 62-year-old man with genetically diagnosed SMA Type 3 with childhood onset. He had a high-amplitude MUP of 10 mV on nEMG of the APB muscle. Patient 2 was a 51-year-old man with genetically diagnosed SBMA with onset in his 30 s.

Magnetic field measurements, signal processing and current estimation were performed as described above. The estimated current distribution was superimposed on an X-ray image based on the marker coil positions that were acquired by both X-ray image and magnetic field measurement.

In the spatial filtering method, the electric current at any point for any direction can be estimated from the magnetic field. Therefore, it is possible to plot the changes in current intensity in any direction at any point over time as a waveform, as if there were virtual electrodes. In this experiment, virtual electrodes were placed to represent the time course of the current on the muscle fibre and the current flowing towards the muscle fibre.

### [Experiment 2] influence of the distance between the sensor array and muscle on motor unit size evaluation

To assess whether the evaluation of MU size was consistent and was not affected by the distance from the sensor array to the muscle, we performed additional measurements. This experiment involved five healthy individuals who participated in Experiment 1 (four men and one woman; 30–43 years of age; median age, 35 years). Magnetic measurements were performed with a stack of paper as a non-magnetic spacer with adjustable thickness to vary the distance between the sensors and the hand. The thickness was adjusted approximately every 5 mm from 0 to 20 mm. Signal processing and current estimation were performed as described above. The estimated current distribution was superimposed on the photograph taken in each measurement position instead of X-rays to reduce radiation exposure from repeated magnetic field measurements.

## Statistical analysis

Statistical analysis was conducted for Experiment 1 and Experiment 2. In Experiment 1, the intraclass correlation coefficient(2,1) and its 95% confidence interval were estimated to assess the consistency between the estimated current and evaluation based on electrical potential. For this analysis, a two-way random-effects model, assuming absolute agreement and single measurements, was used to analyse the onset latencies of each waveform in healthy individuals. This analysis was performed using the intraclass correlation coefficient() function within the *psych* package in R (version 4.4.2). In Experiment 2, we used a generalized linear mixed-effects model (GLMM) to examine whether the distance affected the evaluation of MU size. The log link function was used, and the error was assumed to follow a gamma distribution because this index has a continuous positive value. The model included the distance from the sensor array to the muscle as a fixed effect and the intercept as a random effect. The coefficient, 95% confidence interval and *P*-value for the fixed effect were estimated using the GLMM. The GLMM was performed with the use of MATLAB software, version R2020b.

### Standard protocol approvals, registration and participant consent

This study was approved by Tokyo Medical and Dental University Hospital, Clinical Research Centre (Approval No.: R2018-014) and conducted in accordance with the Declaration of Helsinki. Written informed consent was obtained from all participants.

## Results

### [Experiment 1] analysis of the motor unit activity of abductor pollicis brevis muscle

The magnetic field signals induced by MU activities were successfully detected from 12 MUs of the APB muscle from the eight healthy individuals and from two MUs from the two patients with SMA and SBMA.

**Figure 3:**
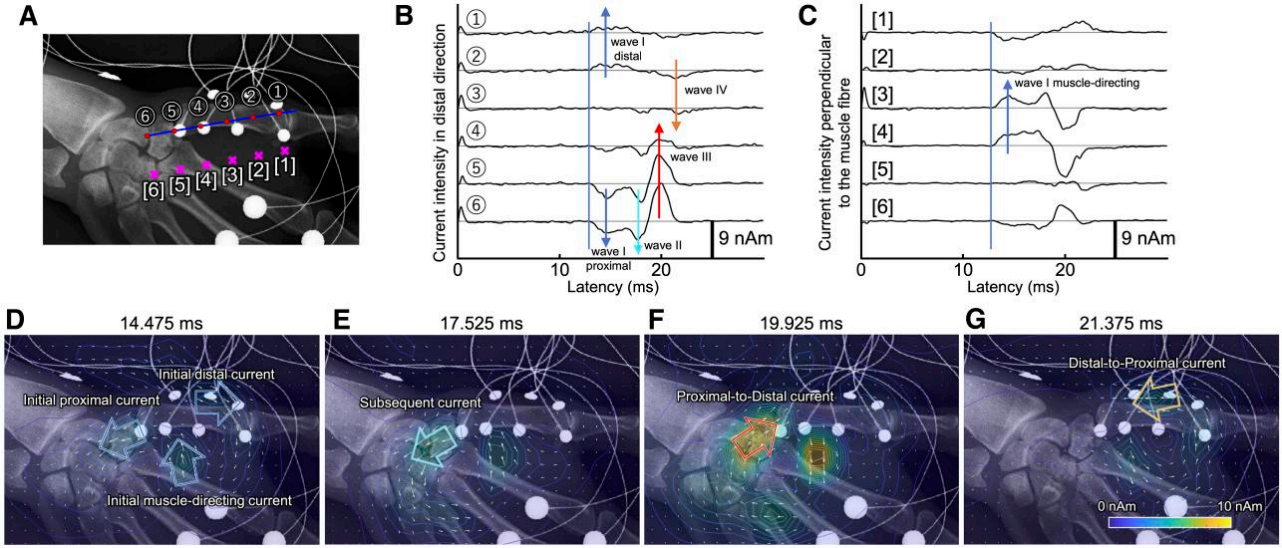
**Waveforms and time course of the current distributions of exemplary motor unit activity in a healthy individual.** (**A**) Positions of the virtual electrodes (dots labelled by circled numbers) placed at 10-mm intervals on the straight line (blue line) connecting the muscle origin and insertion. The position is also shown of the virtual electrode (magenta x-marks labelled by square bracketed numbers) placed on the ulnar side 15-mm perpendicularly from the straight line. (**B**) Waveforms of the current intensity at the virtual electrodes (red dot in (**A**)) (upwards is from the proximal to the distal direction). (**C**) Waveforms of the current intensity towards the muscle fibre at the virtual electrodes (magenta x-mark in (**A**)) (upwards is towards the muscle fibre). (**D**), (**E**), (**F**) and (**G**) show the estimated current distribution as a pseudo-colour map (the colour corresponds to the intensity of the current) with a quiver plot (the white arrows indicate the direction and relative intensity of the current) at 14.475, 17.525, 19.925 and 21.375 ms, respectively. Current towards the muscle and currents to the proximal and distal directions from the middle of the muscle were observed in (**D**). We refer to these currents as ‘Initial muscle-directing current’, ‘Initial proximal current’ and ‘Initial distal current’, respectively. Subsequently, current to the proximal direction flowed in the proximal part of the muscle in (**E**). We call this current ‘Subsequent current’. Current in the distal direction flowed from the proximal part of the muscle in (**F**), while current in the proximal direction flowed from the distal part of the muscle in (**G**). We refer to these currents as ‘Proximal-to-Distal current’ and ‘Distal-to-Proximal current’, respectively.

First, we show the time course of exemplary MU activity in a healthy individual. The magnetic fields evoked by the electric stimulation were strong near the APB muscle (Supplementary Fig. 1). The magnetic field waveforms corresponding to the exemplary MU activity at each sensor are shown at their sensor location in the hand X-ray image. Then, we show the current activity calculated from these magnetic fields as a virtual electrode waveform. Figure 3A shows the virtual electrodes set at 10-mm intervals along the straight line connecting the muscle origin and insertion: the scaphoid bone and base of the proximal phalanx of the thumb (red dots labelled by circled numbers). On these virtual electrodes, waveforms were calculated with the distal component upwards and the proximal component downwards (Fig. 3B). Additional virtual electrodes were placed on the ulnar side, 15 mm away from the straight line (magenta x-marks labelled by square bracketed numbers in Fig. 3A). Waveforms on these electrodes were then calculated with the perpendicular component to the muscle fibre as upwards (Fig. 3C). We explain the waveforms by labelling four typical peaks as waves I through IV. Currents started flowing with a latency of 13.0 ms (blue line in Fig. 3B and C). Initially, the wave was downwards in the proximal electrodes placed on the muscle fibre and upwards in the distal electrodes (blue arrows in Fig. 3B). In the waveform representing the muscle-directing current, the wave was upwards in the middle electrodes (blue arrow in Fig. 3C). We refer to these currents as ‘Wave I proximal’, ‘Wave I distal’ and ‘Wave I muscle-directing’, respectively and collectively refer to them as ‘Wave I’. Subsequently, the waveforms in the proximal electrodes placed on the muscle fibre had a second downward peak (light blue arrow in Fig. 3B). We call this wave ‘Wave II’. Later, an upward wave appeared in the proximal electrodes (‘Wave III’), while a downward wave occurred in the distal electrodes (‘Wave IV’) (red arrow and orange arrow in Fig. 3B).

These waves can also be visualized as current distribution maps on the X-ray image taken in the measurement position. At a latency of between about 13.5 and 17 ms, current towards the muscle and currents to the proximal and distal directions from the middle of the muscle were observed (Fig. 3D). We refer to these currents as ‘Initial muscle-directing current’, ‘Initial proximal current’ and ‘Initial distal current’, respectively and collectively refer to them as ‘Initial current’. These currents correspond to ‘Wave I’ in the waveform. Subsequently, current to the proximal direction flowed in the proximal part of the muscle at a latency of between about 17 and 18.5 ms (Fig. 3E). We call this current ‘Subsequent current’, and this current corresponds to ‘Wave II’ in the waveform. Current in the distal direction flowed from the proximal part of the muscle at a latency of between about 19 and 20.5 ms (Fig. 3F), while current in the proximal direction flowed from the distal part of the muscle at a latency of between about 21 and 23 ms (Fig. 3G). We refer to these currents as ‘Proximal-to-Distal current’ and ‘Distal-to-Proximal current’ and these currents correspond to ‘Wave III’ and ‘Wave IV’ in the waveform, respectively. This MU activity is also shown as a video (Video 1). These waves or current distributions appeared discretely, with no apparent propagation of activity in the current waveforms or in the time course of the current distribution.

Next, we show the characteristics of the 12 MUs of the APB muscle of the healthy individuals. Initial current, Subsequent current, Proximal-to-Distal current and Distal-to-Proximal current, which were observed in the exemplary MU, were seen in the other MUs (Supplementary Fig. 2). Even in these MUs, no propagation of activity was evident. Occurrence rates, ranges of intensities and ranges of times from onset to the maximum of these currents are summarized in Table 1. Because onset latency can depend on the location of the stimulation and the conduction velocity of the evoked motor neurone, the time from onset was evaluated instead of the time from the stimulus (latency).

**Table 1:** Occurrence rates, intensities and times from current onset to maximum

Current name	Occurrence rate	Range of intensity (nAm)^a^	Range of time from onset to maximum (ms)
Initial muscle-directing	12/12	0.44–9.05	1.10–3.05
Initial proximal	12/12	0.83–10.18	0.80–3.10
Initial distal	11/12	0.36–3.24	0.73–3.38
Subsequent	3/12	2.55–5.33	3.03–4.53
Proximal-to-distal	12/12	2.12–23.49	4.43–8.48
Distal-to-proximal	12/12	0.97–7.97	4.35–9.83

^a^For Initial muscle-directing current, the intensity of the component perpendicular to the muscle fibres and, for Initial proximal and distal current, the intensity of the component parallel to the muscle fibres.

### Initial muscle-directing current

In all MUs, the onset latency in the estimated current waveforms coincided with that in the simultaneously recorded electrical potential waveforms (Supplementary Fig. 3). Using the electrical potential waveform as a reference, the onset latency for the estimated current waveform ranged between −0.2 and 0.35 ms. intraclass correlation coefficient(2,1) was 0.99 (95% confidence interval: 0.96–1.00), indicating excellent reliability.

**Figure 4:**
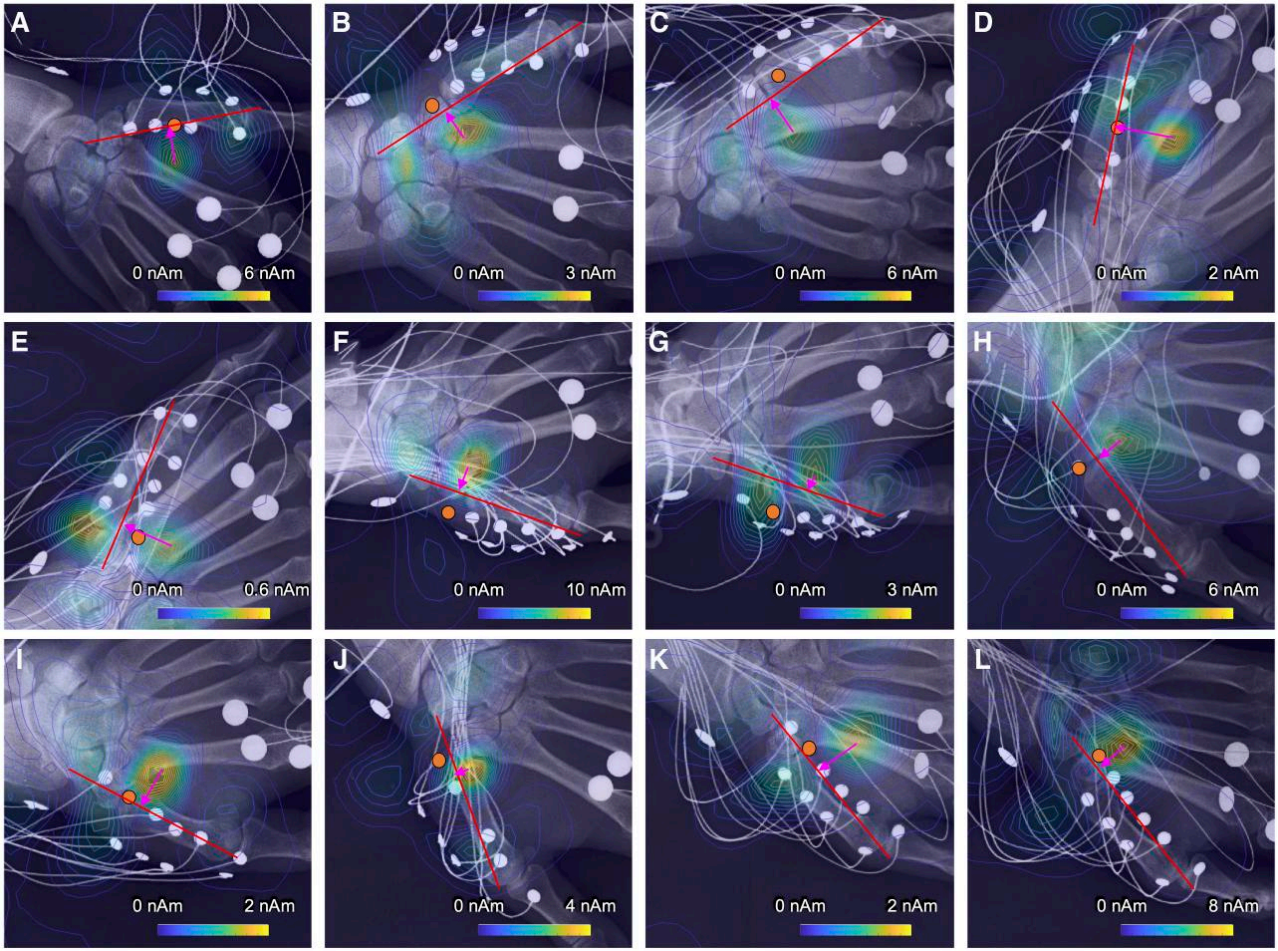
**Initial muscle-directing current and innervation zone position.** (**A–L**) Show contour maps based on the current component perpendicular to the muscle fibre of 12 motor units in healthy individuals. The latencies in these maps are the latencies when the perpendicular component of Initial muscle-directing current reaches its maximum. Orange dots indicate the positions of the innervation zone. The line connects the origin and insertion of the abductor pollicis brevis muscle. A perpendicular line was drawn from the maximum point to the muscle fibre. The foot of the perpendicular line (magenta arrow) was almost compatible with the innervation zone.

Initial muscle-directing current was observed in all MUs. The latency when the current component perpendicular to the muscle fibre reached its maximum was identified (Initial muscle-directing current maximum latency). Figure 4 shows contour maps based on the current component perpendicular to the muscle fibre at that latency. A perpendicular line was drawn from the maximum point to the muscle fibre. The foot of the perpendicular line was almost compatible with the innervation zone. The location of the innervation zone was estimated as the surface potential electrode on the APB muscle that exhibited a waveform with the highest potential amplitude. The time from the onset to the Initial muscle-directing current maximum latency ranged between 1.10 and 3.05 ms. The median was 1.65 ms. The maximum intensity of the perpendicular component of Initial muscle-directing current ranged between 0.44 and 9.05 nAm. The median was 3.29 nAm (Supplementary Figs. 4 and 5 and Supplementary Table 1).

### Initial proximal current and initial distal current

All 12 MUs had an Initial proximal current, and their onset latencies were the same as that of Initial muscle-directing current. One out of the 12 MUs did not have Initial distal current (Supplementary Fig. 2H). In 10 out of the 11 MUs that had both Initial proximal and Initial distal currents, the onset latencies of Initial muscle-directing current, Initial proximal current and Initial distal current were the same. However, in one MU, the onset latency of Initial distal current was 1.25 ms longer than that of Initial muscle-directing current and Initial proximal current (Supplementary Fig. 2B).

### Subsequent current

In 3 of the 12 MUs, Subsequent current was observed (green arrow in Supplementary Fig. 2A, D and J).

### Proximal-to-distal current and distal-to-proximal current

After Initial current or Subsequent current, Proximal-to-Distal current (red arrow in Supplementary Fig. 2) and Distal-to-Proximal current (orange arrow in Supplementary Fig. 2) appeared in all MUs. The order of appearance of these currents was different for each MU (Supplementary Fig. 6), and they appeared twice in two MUs (Supplementary Fig. 2B and J). In most MUs, the maximum intensities of Proximal-to-Distal current were larger than those of Distal-to-Proximal current or the perpendicular component of Initial muscle-directing current (Supplementary Fig. 5).

### Comparison between healthy individuals and patients with spinal muscular atrophy and spinal bulbar muscular atrophy

**Figure 5:**
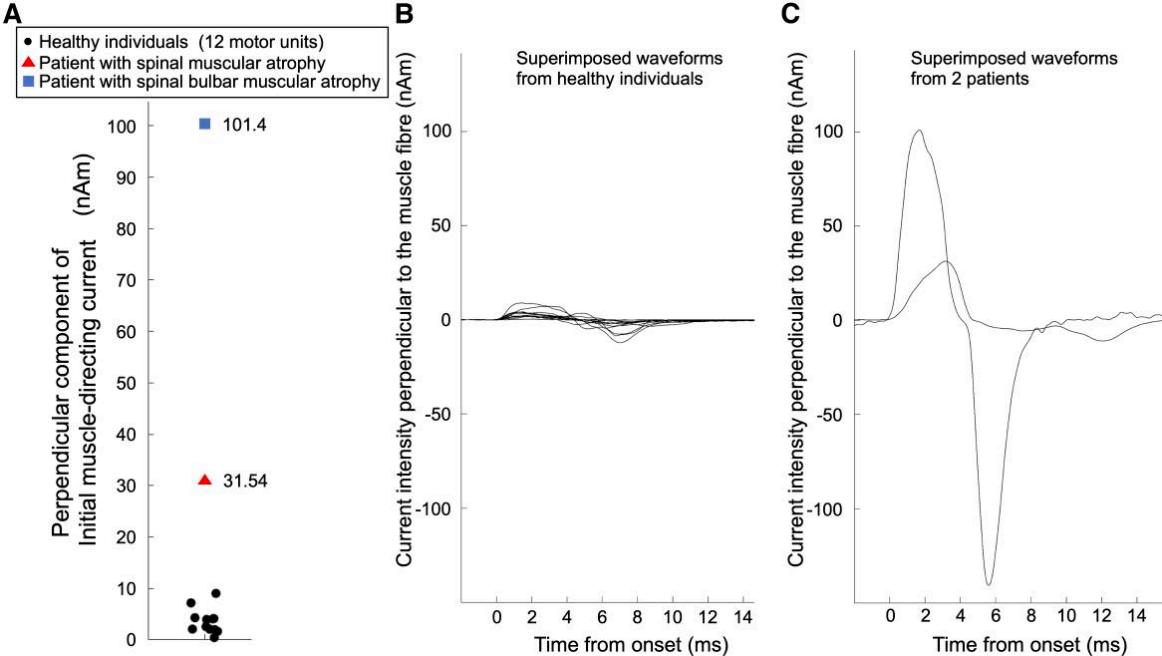
**Comparison of motor unit currents between healthy individuals and patients with spinal muscular atrophy and spinal bulbar muscular atrophy.** (**A**) The intensity of the maximum perpendicular component of Initial muscle-directing current of the 12 motor units from eight healthy individuals (black dots; *n* = 12) and the two motor units from two patients with spinal muscular atrophy (triangle; *n* = 1) and spinal bulbar muscular atrophy (blue rectangle; *n* = 1). (**B**) Superimposed waveforms of the 12 motor units (*n* = 12) from eight healthy individuals. (**C**) Superimposed waveforms of the two motor units (*n* = 2) from two patients. These waveforms show the time course of the estimated current intensity perpendicular to the muscle fibre at the maximum point of the perpendicular component of Initial muscle-directing current. The waveforms are aligned with the onset latency set to 0 ms.

Initial muscle-directing current was also observed in MUs from two patients with SMA and SBMA. The maxima of the Initial muscle-directing current component perpendicular to the muscle fibres were 31.5 nAm (Patient 1) and 101.4 nAm (Patient 2), which were larger than the range observed in healthy individuals (0.44–9.05 nAm) (Fig. 5A–C).

### [Experiment 2] influence of the distance between the sensor array and muscle on motor unit size evaluation

Five activities of MUs of the APB muscle from five healthy individuals were measured. Supplementary Fig. 7 shows the relationship between the maximum perpendicular component of Initial muscle-directing current as an evaluation of MU size and the distance from the sensor array using the regression curve estimated by the GLMM. Although a change in the distance from the sensor array had a statistically significant effect on the intensity of the maximum perpendicular component of Initial muscle-directing current (*P* = 0.0092), it was estimated to weaken by only 5.5% for every 5 mm from the sensor. The estimated regression curve was *y* = exp(−0.0113*x* + 1.535), where *x* is the distance from the sensor array to the muscle (mm) and *y* is the intensity of the maximum perpendicular component of Initial muscle-directing current (nAm). The 95% confidence interval for the coefficient of *x* was [−0.0195, −0.0031].

## Discussion

The magnetic fields of single MUs of the APB muscle were successfully measured in healthy individuals and patients with SMA and SBMA. Using the magnetic field distribution, it was possible to estimate the current distribution from a muscle in the hand, which has a complex configuration and conductivity distribution.

In this study, we applied the spatial filtering method. This method can estimate the current distribution from the magnetic field, even if the configuration or conductivity distribution of the conductor is unknown, and does not require an a priori assumption of how many source currents are present.^33,51^ As another method for estimating currents from magnetic fields, the equivalent current dipole method has been clinically applied to estimate the epileptic focus.^52-57^ Masuda *et al.*^23^ also used this method to calculate the intensity of the MU activity current. The equivalent current dipole method assumes a single current element as the source current. The position, intensity and direction of the source are determined to minimize the difference between the observed magnetic field and the magnetic field associated with that source current and its volume current.^58^ The current due to the electromotive force impressed by biological activity is called the source current (also called the primary current). The source current generates a secondary current (also called the volume current or the recurrent current) in the surrounding area to satisfy the law of conservation of electric charge.^24,33^ The flow of the volume current depends on the configuration and conductivity distribution of the volume conductor. For conductors that can assume a sphere or semi-infinite plane, it is possible to calculate the volume current and associated magnetic field.^24^ However, for complex shapes, it is difficult to calculate them analytically. If the source current is assumed to be multiple or continuous, the assumption of the equivalent current dipole method does not hold. Although multiple current sources can be assumed and fitted to the observed magnetic field, the number of current sources must be assumed a priori before the calculation. In peripheral nerves, the activity is considered to have length^44^ and to form a quadrupole as a whole,^59,60^ rather than activity at a single point. Muscle electrical activity is not qualitatively different from that of the nerve.^61,62^ Therefore, before calculating the equivalent current dipole method for muscle activity, it is necessary to hypothesize the shape of the source current. In addition, how the volume current flows around the muscle must be known. In contrast, for current estimation using the spatial filtering method, no distinction is made between the source current and the volume current. At many estimation points, the intensity and direction are calculated as current elements according to the location.^58^ The spatial filtering method has also been used for magnetoencephalography,^63^ and we have also used this method to evaluate spinal cord and peripheral nerve activity.^34-46^

We speculate that Initial muscle-directing current reflects currents flowing towards the muscle fibres due to activity near the neuromuscular junction. To explain this, we provide some physiological background. Differences in ion concentrations between the inside and outside of the cell membrane and the resulting osmotic pressure create a flow of ions. The opening and closing of ion channels alter ion permeability, and the membrane behaves like a variable power source.^64^ Secretion of acetylcholine from the alpha motor neurone terminals opens acetylcholine-gated ion channels and creates a local positive potential change inside the muscle fibre membrane, called the end-plate potential. In turn, this end-plate potential opens voltage-gated sodium channels.^62^ This behaves as an electromotive force, generating the source current. Current from the excited membrane flows bidirectionally along the fibre. It then passes through the extracellular tissue to return to the excited membrane.^65^ This current is the volume current. Initial muscle-directing current identified in this study was observed at the beginning of the activity and flowed towards the innervation zone. At the same time, Initial proximal and distal currents were observed in most MUs. These results suggest that they are caused by the activity near the neuromuscular junction. This current, which triggers the subsequent motor unit activity, has not been visualized by previous methods but can now be evaluated with this method. Similar to the current distribution observed around the muscle in this study, our group has visualized the currents of peripheral nerves and spinal cords that flow perpendicular to the fibre, parallel to it bidirectionally and return from the environment.^36-41,43^

Initial muscle-directing current estimated in this study had its strongest point away from the muscle fibre. However, the muscle-directing current flowing from the excited membrane into the muscle fibre is theoretically considered to be strongest at the boundary between the muscle fibre and its surrounding tissue. The reason for this inconsistency may be that the current is estimated from the magnetic field. Because currents flow into the muscle fibres from both radial and ulnar sides in opposite directions, the magnetic field distributions associated with the currents cancel each other out. Therefore, the current estimated from the magnetic field also becomes smaller. However, at a distance from the muscle fibre, the currents are oriented in the same direction, so the cancellation becomes weaker and the estimated current becomes larger. In addition, the current from the ulnar side was more easily visible than that from the radial side. This is thought to occur because more volume current flows on the ulnar side, as there are fewer conductors on the radial side.

In this study, we were unable to observe the propagation of excitation from the innervation zone in the muscle activity. In contrast, our studies of peripheral nerves and spinal cords have shown the propagation of excitation.^36,37,39-41^ This could be because, unlike neural activity, excitation in muscle activity propagates in both directions. Neural and muscular activity forms quadrupoles. In muscular activity, excitation propagates bidirectionally, so there are two quadrupoles. The balance of the proximity of the two quadrupoles and the distance from the muscle to the sensor array results in a single quadrupole magnetic field distribution pattern, even with two quadrupoles. This has been shown in a simulation study.^61^ Therefore, the two excitations may not be separated in the current distribution estimated from a magnetic field observed at a distance.

In all MUs, after Initial current, currents flowed from both ends of the muscle towards the muscle belly (Proximal-to-Distal current and Distal-to-Proximal current). These currents are thought to be associated with activity near the tendon at both ends. Tendons have a lower conductivity than muscles.^66^ Thus, when the excitation reaches the vicinity of the tendon, the volume current flows more towards the muscle belly and less towards the tendon. Furthermore, at this time, the magnetic field becomes larger because the magnetic fields are less cancelled by the currents in opposite directions,^61^ so the estimated current is also larger. The order of appearance of these currents differs in MUs, perhaps because of differences in the distance from the innervation zone to the tendon and/or the conduction velocity of the muscle action potential. In two MUs, Proximal-to-Distal or Distal-to-Proximal current was observed twice. This could be attributed to the fact that muscle fibres within a MU, extending across various muscle bundles, might terminate at different locations.^67^ This could result in multiple excitations near the tendon, causing the current to appear twice.

The effect of tendons may also be related to Initial current and Subsequent current. No Initial distal current was observed in one MU. In this MU, the innervation zone may be biased towards the distal tendon, which may have weakened the volume current flowing distally. In three MUs, Subsequent current was observed. This might be the result of the activity near the tendon superimposed on the activity near the innervation zone.

While Masuda *et al.*^23^ evaluated MU size from the activity that presented the largest magnetic field in which excitation was expected to reach the vicinity of the distal tendon (corresponding to Distal-to-Proximal current in our study), we consider the Initial muscle-directing current component perpendicular to the muscle fibre to be a preferable indicator for quantifying MU size. We believe that it is better to evaluate MU size by using Initial muscle-directing current for several reasons. First, Initial muscle-directing current is considered to be the current associated with activity near the neuromuscular junction and has a clearer physiological interpretation than other currents. Second, Initial muscle-directing current is considered to more directly reflect the MU size, with less influence from factors such as those discussed below, although it is constantly attenuated by current from the opposite side. On the other hand, the intensity of the other currents may be influenced by factors other than the MU size. For example, changes in the conductivity of the tendon can affect the intensity of the magnetic field associated with Proximal-to-Distal and Distal-to-Proximal currents. In addition, if these occur simultaneously, the observed magnetic fields weaken each other because they are in opposite directions. Finally, Initial muscle-directing current appears only once and can be evaluated in all MUs. Proximal-to-Distal current or Distal-to-Proximal current appeared twice in two MUs and is therefore difficult to evaluate. In addition, Subsequent current does not always appear and is therefore unsuitable for evaluation. As a side note, we extracted and evaluated only the perpendicular component of Initial muscle-directing current. This is due to the presence of MUs in which the Initial muscle-directing current was continuous with Initial proximal current or Initial distal current in the current distribution map (data not shown).

According to the results of Experiment 2, the perpendicular component of Initial muscle-directing current as the MU size was estimated to be almost constant even when the distance from the sensor array to the muscle was changed. Even though the MU was moved 5 mm away from the sensor array, the estimated current value only decreased by 5%. On the other hand, in Surface electromyography, the amplitude of the MUP changes by about 10 times when the depth of the MU is 3 mm from the surface versus 10 mm.^68^ Thus, our method is much less affected by distance from the sensor than surface electromyography. Furthermore, because the MU size was estimated to be much larger in SMA and SBMA patients than in healthy individuals, the attenuation of the evaluation of MU size due to the distance was considered minimal. We believe that the estimated current decreased with distance, albeit slightly, due to the effect of the current on the opposite side. In other words, the attenuation of the magnetic field is not only due to the distance, but also due to cancellation by the counter-directional magnetic field generated by the current on the opposite side. The magnetic field attenuation caused by the distance from the sensor array can be corrected by moving the estimation area farther away. However, the attenuation caused by cancellation by opposite currents cannot be corrected.

## Conclusion

Magnetic field measurements of the activities of single MUs of the APB muscle and current estimation using spatial filtering showed current towards the innervation zone in all MUs immediately after the start of activities. This Initial muscle-directing current was much larger in patients with SMA and SBMA than in healthy individuals. Initial muscle-directing current is considered to reflect the activity near the neuromuscular junction and can be an index of MU size. In contrast to other techniques in use today, this novel and non-invasive method can evaluate the MU size with little influence of distance. We believe that magnetomyography has the potential to supersede nEMG in assessing motor unit size.

## Supplementary Material

fcaf294_Supplementary_Data

## Data Availability

The data supporting the findings of this study are available from the corresponding author upon reasonable request.
