# Supplementary material for: Magnetomyographic evaluation of motor unit size that is robust to changes in distance to sensor
Source: Brain Commun. 2025 Aug 20;7(4):fcaf294. doi: 10.1093/braincomms/fcaf294 (PMC12451695; doi:10.1093/braincomms/fcaf294)
Supplement: fcaf294_Supplementary_Data [file fcaf294_Supplementary_Data.pdf]

SUPPLEMENTARY MATERIAL

Magnetomyographic evaluation of motor unit size that is robust to changes in distance to sensor

Tai Otani*, Miho Akaza*, Shigenori Kawabata, Hirokazu Natsui, Taishi Watanabe, Yuki Miyano, Ryoichi Hanazawa, Yoshiaki Adachi, Kensuke Sekihara, Tadashi Kanouchi and Takanori Yokota

**Tai Otani and Miho Akaza contributed equally to this work.*

Table of contents

Supplementary Table

- Supplementary Table 1. Maximum intensity of perpendicular component of Initial muscle-directing current and its time from onset

Supplementary Figures

- Supplementary Fig. 1. Magnetic fields of exemplary motor unit activity
- Supplementary Fig. 2. Virtual electrode position and current waveform for 12 motor units
- Supplementary Fig. 3. Comparison of the onset latency of the estimated current waveform and the potential waveform
- Supplementary Fig. 4. Maximum intensity of the perpendicular component of Initial muscle-directing current and its time from onset
- Supplementary Fig. 5. Maximum intensity of the perpendicular component of Initial muscle-directing current, Proximal-to-Distal current and Distal-to-Proximal current
- Supplementary Fig. 6. Time from the onset to the maximum Proximal-to-Distal current and Distal-to-Proximal current
- Supplementary Fig. 7. Relationship between the maximum intensities of the perpendicular component of Initial muscle-directing current and the distance from the sensor array to the muscle

Supplementary Table 1. Maximum intensity of perpendicular component of Initial muscle-directing current and its time from onset

Motor unit	Perpendicular component of Initial muscle-directing current (nAm)	Time from onset to maximum perpendicular component of Initial muscle-directing current (ms)
A	4.32	1.475
B	2.59	2.325
C	3.98	2.550
D	2.00	1.875
E	0.44	2.125
F	9.05	1.450
G	2.10	1.725
H	4.10	2.400
I	2.11	1.350
J	4.16	1.150
K	1.66	1.575
L	7.20	3.050

Supplementary Fig. 1. Magnetic fields of exemplary motor unit activity

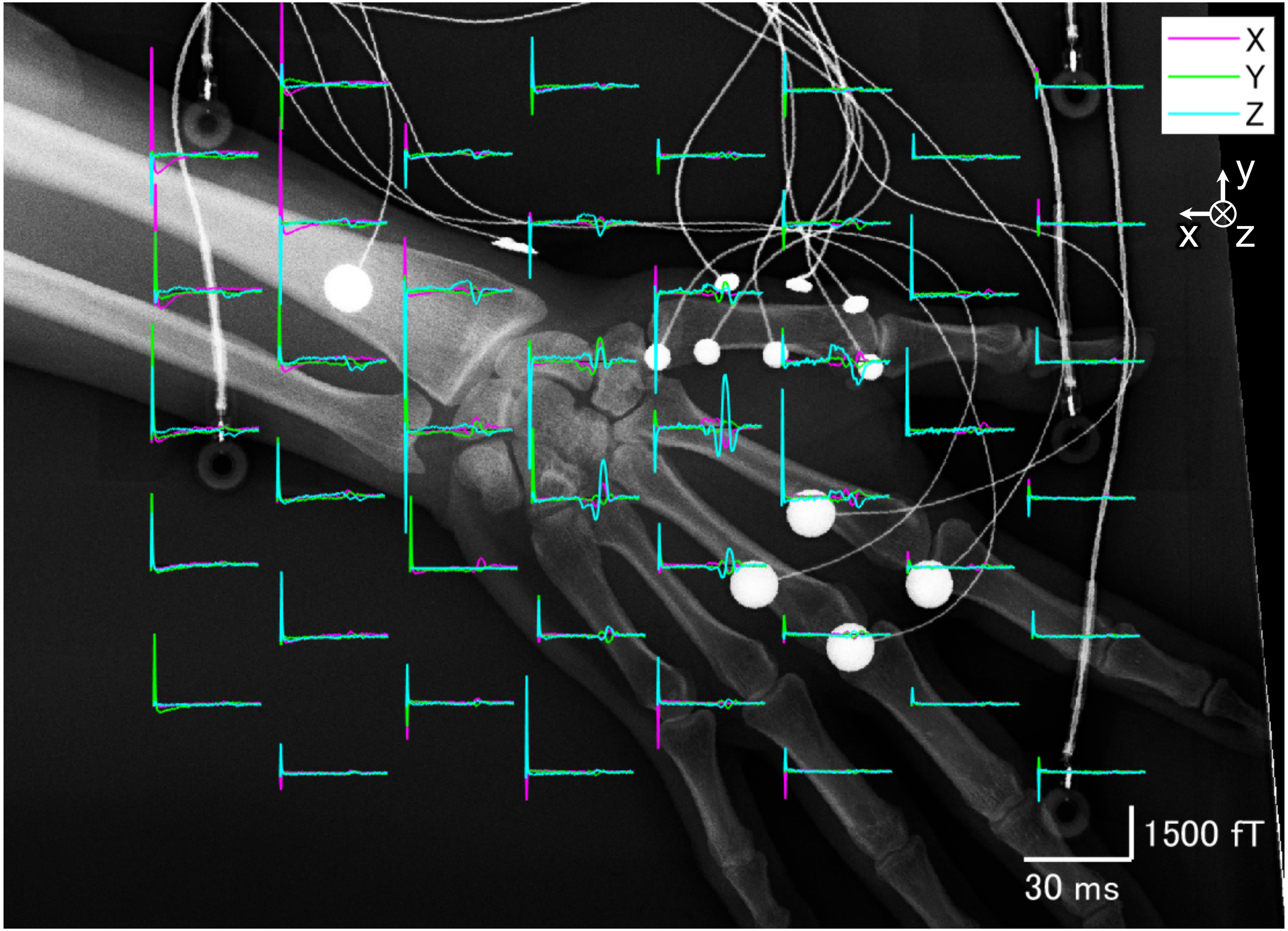

The figure shows the three-directional magnetic signals recorded by each sensor after the stimulation superimposed on an X-ray image of the hand. Viewed from the dorsal side of the hand, the sensor array was under the palmar side of the hand. Magenta traces are magnetic fields in the right to left direction (X component). Green traces are magnetic fields in the bottom to the top direction (Y component). Cyan traces are magnetic fields in the dorsal to palmar direction (Z component).

Supplementary Fig. 2. Virtual electrode position and current waveform for 12 motor units

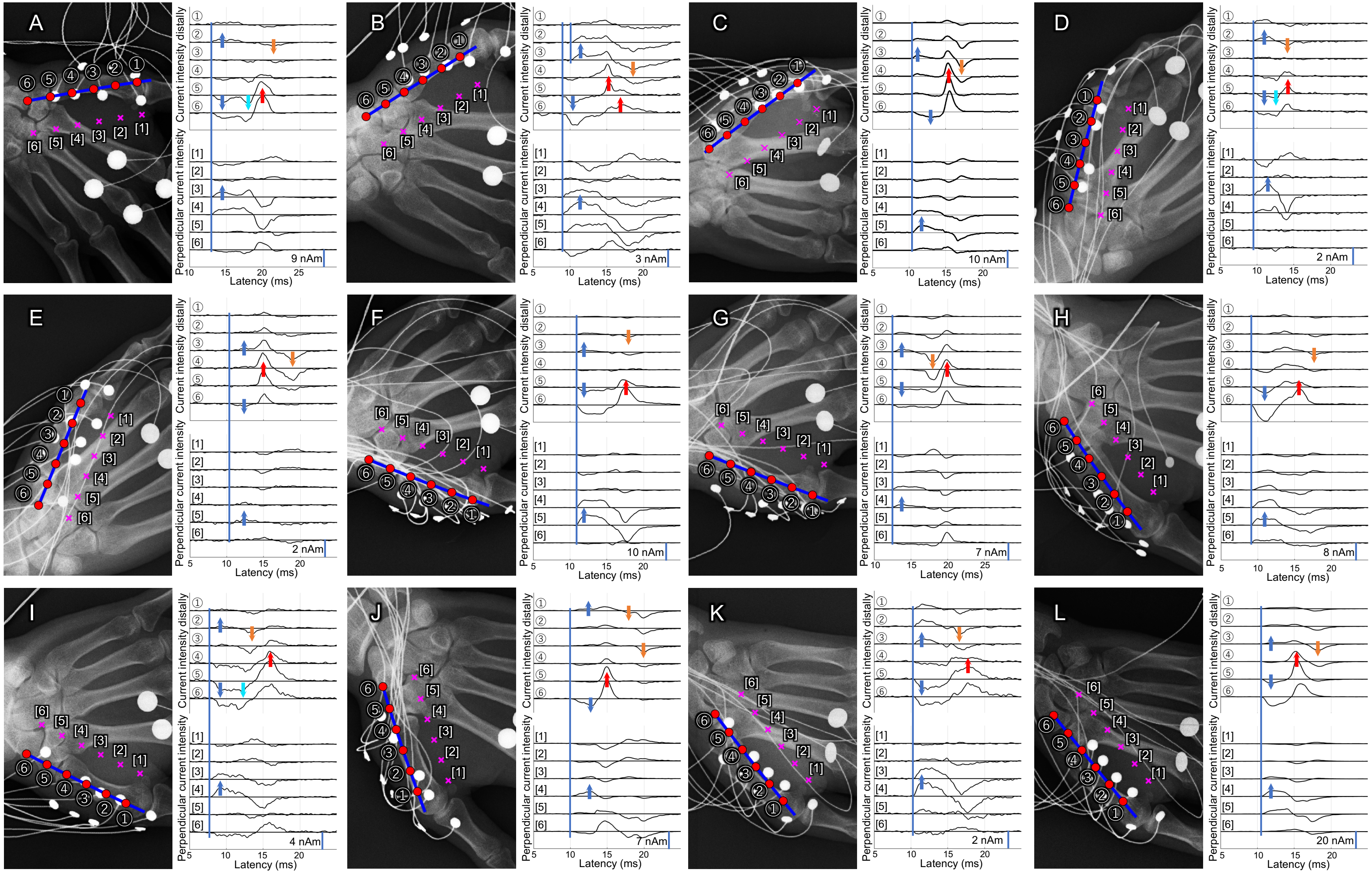

Virtual electrode positions and current waveforms for 12 motor units (MUs) are shown. The positions of the virtual electrodes (red dots labelled by circled numbers) placed at 10-mm intervals on the straight line (blue line) connecting the muscle origin and insertion are shown on the left. The positions are also shown of the virtual electrodes (magenta x-marks labelled by square bracketed numbers) placed on the ulnar side 15-mm perpendicularly from the line. Waveforms on the upper right show the time course of the current intensity at the virtual electrodes (red dots) (upwards is from the proximal to the distal direction). Waveforms on the lower right show the time course of the current intensity towards the muscle fibre at the virtual electrodes (magenta x-marks) (upwards is towards the muscle fibre). Blue arrows indicate Initial current. Light blue arrows indicate Subsequent current. Red arrows indicate Proximal-to-Distal current. Orange arrows indicate Distal-to-Proximal current.

Supplementary Fig. 3. Comparison of the onset latency of the estimated current waveform and the potential waveform

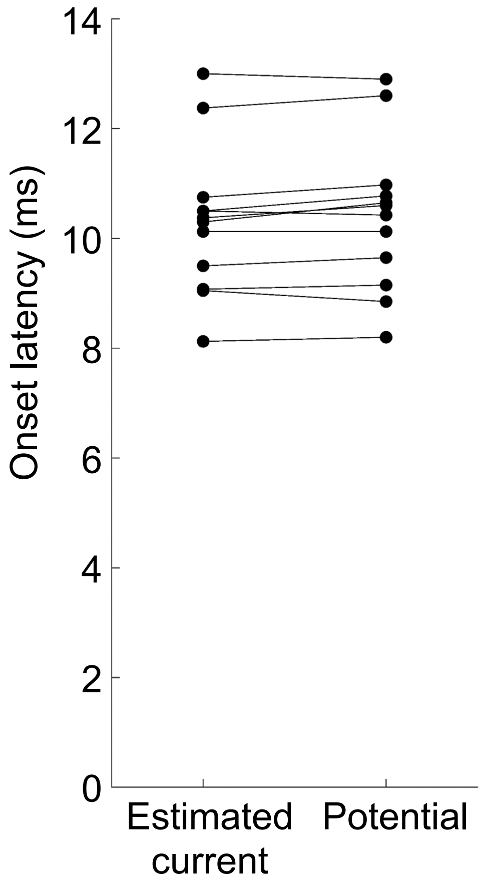

Dots indicate the individual onset latencies of the estimated current waveforms and the potential waveforms from12 motor units (n = 12). These onset latencies coincided with each other. The intraclass correlation coefficient (ICC(2,1)) was 0.99 (95% confidence interval: 0.96–1.00), indicating excellent reliability.

Supplementary Fig. 4. Maximum intensity of the perpendicular component of Initial muscle-directing current and its time from onset

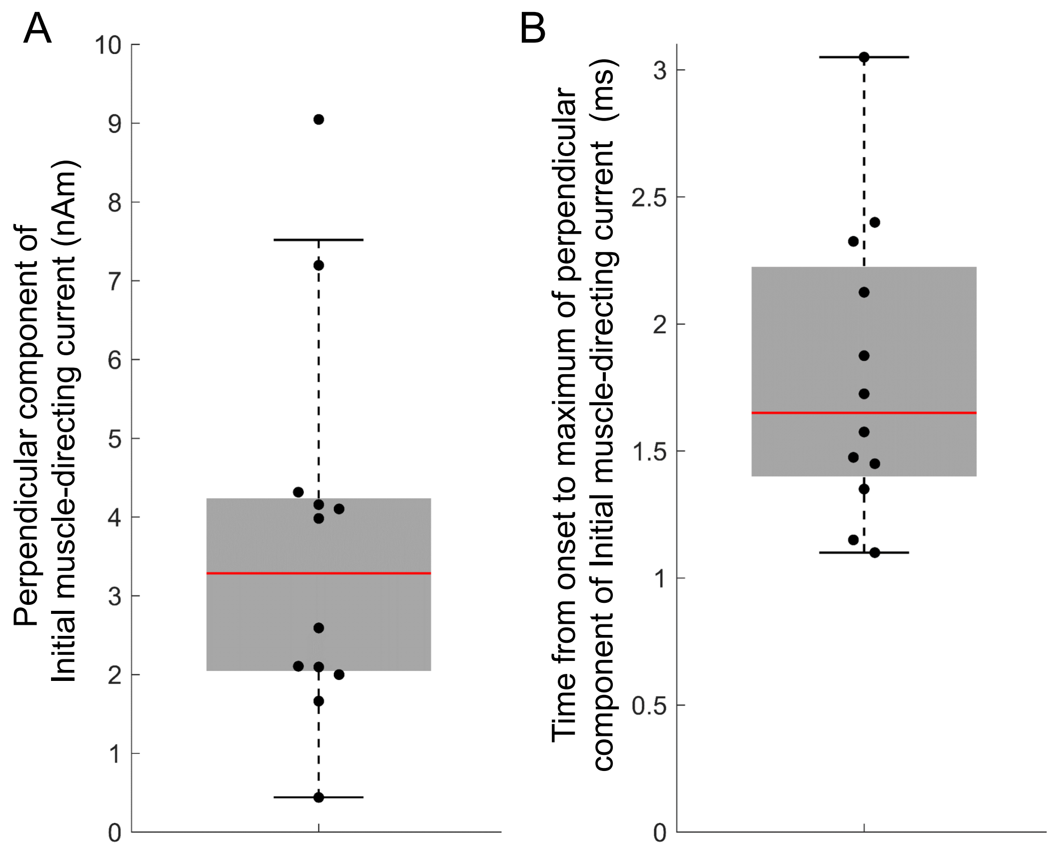

(A) A box-and-whisker plot shows the maximum intensity of the perpendicular component of Initial muscle-directing current of 12 motor units (n = 12) from healthy individuals. (B) A box-and-whisker plot shows time from onset to the maximum perpendicular component of Initial muscle-directing current of 12 motor units (n = 12) from healthy individuals. Each dot represents the value from one motor unit.

Supplementary Fig. 5. Maximum intensity of the perpendicular component of Initial muscle-directing current, Proximal-to-Distal current and Distal-to-Proximal current

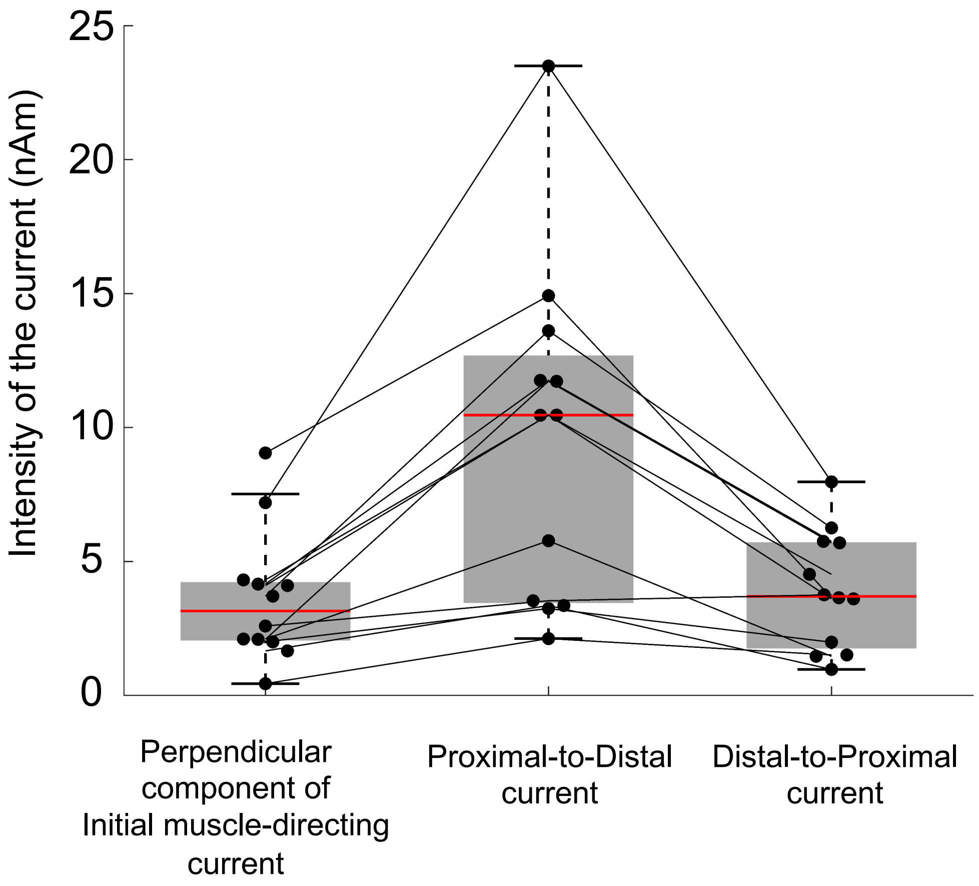

A box-and-whisker plot shows the maximum intensities of the perpendicular component of Initial muscle-directing current, Proximal-to-Distal current and Distal-to-Proximal current of 12 motor units (n = 12) from healthy individuals. Each dot represents the value from one motor unit. Dots from the same motor unit are connected by lines to show their relationship. In most MUs, the maximum intensities of the Proximal-to-Distal current are larger than those of the Distal-to-Proximal current or the perpendicular component of Initial muscle-directing current.

Supplementary Fig. 6. Time from the onset to the maximum Proximal-to-Distal current and Distal-to-Proximal current

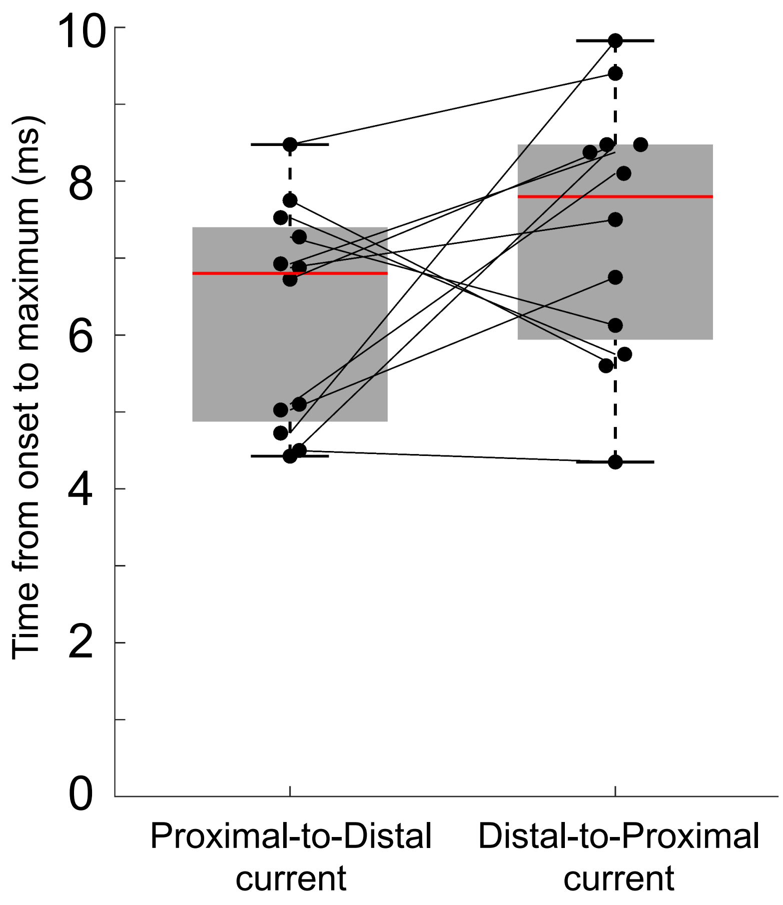

A box-and-whisker plot shows times from the onset to the maximum Proximal-to-Distal current and Distal-to-Proximal current of 12 motor units (n = 12) from healthy individuals. Each dot represents the value from one motor unit. Dots from the same motor unit are connected by lines to show their relationship. Some motor units exhibit Proximal-to-Distal current first, while others exhibit Distal-to-Proximal current first.

Supplementary Fig. 7. Relationship between the maximum intensities of the perpendicular component of Initial muscle-directing current and the distance from the sensor array to the muscle

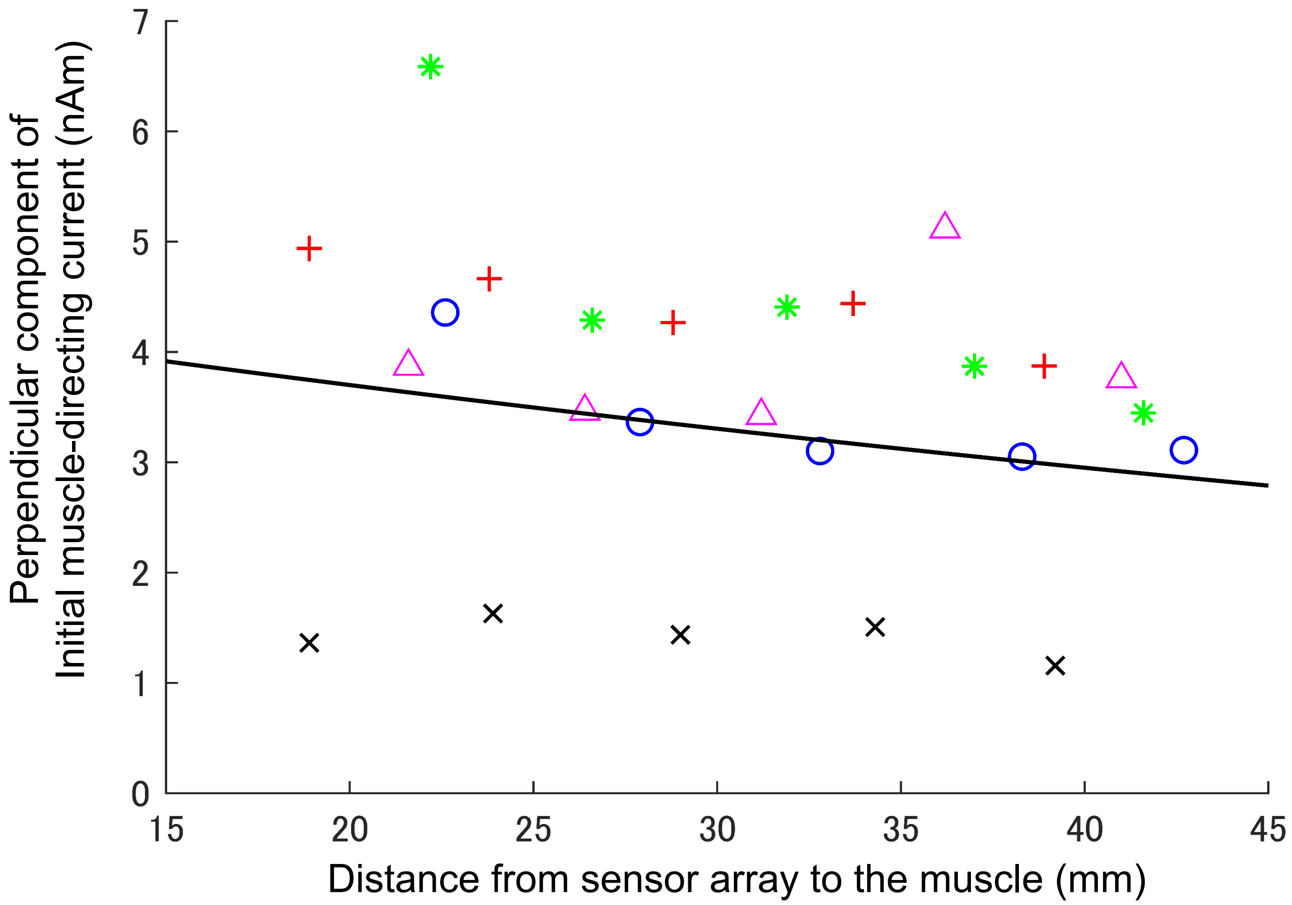

The figure shows the relationship between the maximum intensities of the perpendicular component of Initial muscle-directing current of five motor units (n = 5) and the distance from the sensor array to the muscle. For each motor unit, measurements were obtained at five different distances, resulting in five data points per motor unit. Values from the same motor unit are shown in the same colour and shape. The regression curve estimated by the generalized linear mixed model is shown as a black curve. The curve is $y = \exp(-0.0113x + 1.535)$, where x is the distance from the sensor array to the muscle [mm] and y is the intensity of the maximum perpendicular component of Initial muscle-directing current [nAm]. The 95% confidence interval for the coefficient of x is [−0.0195, −0.0031].
